# Tumor patients’ fears and worries and perceived changes of specific attitudes, perceptions and behaviors due to the COVID-19 pandemic are still relevant

**DOI:** 10.1007/s00432-021-03573-y

**Published:** 2021-03-06

**Authors:** Arndt Büssing, Daniela Rodrigues Recchia, Jutta Hübner, Stefanie Walter, Judith Büntzel, Jens Büntzel

**Affiliations:** 1 Quality of Life, Spirituality and Coping, Witten/Herdecke University, Herdecke, Germany; 2 Chair of Research Methods and Statistics in Psychology, Faculty of Health, Witten/Herdecke University, 58448 Witten, Germany; 3 Klinik Für Innere Medizin, Universitätsklinikum II, University Clinic Jena, Jena, Germany; 4 Bundesverband der Kehlkopfoperierten E. V, Bonn, Germany; 5 Department of Hematology and Medical Oncology, Universitätsmedizin, Göttingen, Germany; 6 Department of Otolaryngology, Palliative Care Unit, Südharz Klinikum Nordhausen, Nordhausen, Germany; 7 Working Group “Prevention and Integrative Oncology” (PRIO) in the German Cancer Society, Berlin, Germany

**Keywords:** Tumor patients, Corona pandemic, COVID-19, Wellbeing, Change of attitudes, Spirituality

## Abstract

**Objective:**

During the COVID-19 pandemic, tumor patients not only perceived fears and worries but were experiencing also positive changes as the perception of nature and silence, moments of wondering awe, and more intense relationships. We intended to analyze whether these perceptions may differ between patients from waves 1 and 2 of the pandemic.

**Methods:**

Cross-sectional study at two time periods (May to June, sample 1) and September to November 2020 (sample 2) with standardized questionnaires (i.e. WHO-5, MLQ, PCQ-12).

**Results:**

Patients from sample 1 (*n* = 292) and sample 2 (*n* = 221) did not differ with respect to gender, age, partner or tumor status. Most are still “irritated by statements about danger and course of the infection” (58%) and are “worrying to be infected and to have complicated course of disease” (55%). Neither their well-being nor meaning in life nor fears and worries were significantly different. In sample 2 patients, Worrying reflections and loneliness scored significantly lower, while their Perception of nature and silence was lower in trend only; more intense relationships are still relevant. Moments of wondering awe and religious trust were perceived less often during wave 2. Particularly religious patients scored stronger for Perception of nature and silence and Worrying reflections and loneliness.

**Conclusion:**

Oncologists/psychologist have to know that patients’ situation has not changed within the time of pandemic and that they still require information, close support and encouragement to rely on their resources to cope. Perceived changes are reflecting coping strategies that could be trained to increase patients’ resilience during further pandemic waves.

## Introduction

The COVID-19 pandemic has restricted social life around the world since spring 2020. More cancer patients are waiting for their relevant and necessary treatments (Reichardt et al. 2020). Surveys are describing increasing fears of the patients and enhancing the stress of the health professionals in daily oncology services (Büntzel et al. 2021). Cancer survivors have lost a lot of possibilities of control, rehabilitation, and networking. Patients and survivors are afraid of self-infection, risks for their families and relatives, decreasing treatment chances and fear social isolation (Lou et al. 2020). The German Comprehensive Cancer Centers have registered decreased services in speaking oncology, e.g. psychosocial consultations, psychological accompaniments or contacts to self-help groups were reduced dramatically (Fröhling and Arndt 2020). German cancer self-help activists state that their work in patient advocacy groups is heavily hampered and restricted by the current situation (Bruhns 2020). Similar reports of gynecologic cancer patients described how social resources (e.g. family members at therapy appointments, support groups) were not available due to pandemic restrictions (Moran et al. 2020). Further, persons with cancer, particularly older ones, are a unique group at risk as they are also more susceptible to various infections (due to an often compromised immune system) and at risk of thromboembolic events—a known complication of COVID-19 infection (Palaskas et al. 2020).

Reliable information on COVID-19 and oncological resources are essential to tumor patients in particular. However, instead of a congruent and non-conflicting narrative, patients are subjected to clashing reports about the danger and the course of the corona infection in the public media (Büntzel et al. 2020a, b, 2021; Büssing et al. 2020a). This reflects their insecurity about how to behave. Recently, another study with 195 cancer patients demonstrated that more than half of them did not know how to correctly use supplements for corona protection; most of them received information on COVID-19 related topics via social media or the television (Guven et al. 2020). Therefore, many patients are unsure whether or not to visit hospitals and oncological wards to be treated. They worry about getting infected and subsequently (while belonging to a vulnerable group) to have a complicated course of the disease. Conflicting information and the fear of being infected aggravate their complicated situation (Büntzel et al. 2020a, b, 2021; Büssing et al. 2020a). Instead, patients might require adequate information and reliable resources to cope.

During the first lockdown in Germany March 2020, several used the time of social distance to become more aware of their relations, of nature as an opportunity to relax and to distance from negative thoughts and emotions, and to connect with others via digital media (Büssing et al. 2020b). This can be seen as a matter of reappraisal coping. During the pandemic, a larger survey among German participants revealed their wellbeing was predicted by low perceived burden due to the pandemic and by multidimensional life satisfaction, with further impact of the ability to stop in ‘wondering awe’ in specific situations (as a matter of mindful awareness and perceptive spirituality), by awareness of *Nature/Silence/Contemplation* and by low worrying reflections of live as predictors (Büssing et al. 2020b). Similarly, tumor patients’ perceptions and attitudes were shaped by their fear about an unclear course of the pandemic, the unclear personal risk to be infected, and by experiences of loneliness due to social distancing (Büssing et al. 2020a). This would indicate that the balance of stressors and resources is crucial to cope with the pandemic.

During the following summer months of 2020, the restrictions were widely reversed in Germany and most assumed that their fears were arbitrary or too strong. Several of the general population ignored the recommendations to nevertheless keep distance and to wear protection masks. The fraction of ‘Corona-Denier’ and rule-breakers became louder in several countries of the world (Sweet 2020; Reuters 2020) and organized protest marches to have the right to not wear protection masks—and thereby contributed to spoil persons at risk, but also health care professionals (Mitchell 2021).

Then during September and October 2020, it was clear that the second wave of the COVID-19 pandemic started and will be even stronger than expected, with a dramatic increase of infected persons, hospitalized patients, and persons dying due to COVID-19 infection. At the start of this second phase, we started to enroll tumor patients again and applied the same questionnaire battery, focusing on their fears and worries, wellbeing and burden, but also on perceived changes in attitudes and behaviors due to the pandemic (i.e. perception of nature and silence; worrying reflections and loneliness; interest in spirituality; intense relationships). Aim was to analyze whether or not tumor patients’ perceptions may have changed within time, whether their fears and worries may have decreased and they were able to emotionally adapt to the situation.

## Materials and methods

### Recruitment of patients

The working group “Prevention and Integrative Oncology” (PRIO) in the German Cancer Society (Deutsche Krebsgesellschaft) has initiated studies among tumor patients during the COVID-19 pandemic. For this study, tumor patients were recruited in the same hospitals and wards as in the first wave (Büssing et al. 2020a), mainly in seven West and East German centers (Solingen, Wetzlar, Bielefeld, München, Herne, Nordhausen, Jena), and with the support of the Federal Cancer Self Care Association (Haus der Krebs-Selbsthilfe—Bundesverband e.V.) with its 10 member organizations within a 2 months time span (from September 15 to November 11). All patients were assured confidentially and were informed about the purpose of the study and data protection information at the starting page of the online survey and at page one of the printed version. By filling in the anonymous questionnaire, patients consented to participate. No concrete identifying personal details were recorded to guarantee anonymity. The study was approved by the IRB of Jena University Clinic (#5497-04/18; amendment from May 5, 2020). We followed the ethical principles of the Helsinki convention. As a reference sample we relied on the patients from the first wave of the study (recruiting from May 6 to June 10, 2020) (Büssing et al. 2020a).

### Measures

#### Perception of changes

To assess perceived changes due to the COVID-19 pandemic, we relied on the 12-item short version of the *Perceptions of Change Scale* (Büssing et al. 2020a) which addresses four main topics: (1) Perception of nature and silence (four items: going outdoors more often and perceiving nature more intensely, taking consciously time for silence and enjoy quite times of reflections), (2) Worrying reflections and loneliness (four items: concerned about meaning in life and the lifetime one has, more intense perception of loneliness and feelings of being cut off from life (due to the pandemic restrictions), (3) Interest in spirituality (two items: praying/meditating more than before, more interest in religious/spiritual topics as a strategy to cope), (4) Intense relationship (two items: more intensive perceptions of relationships with partner/family and with friends). The respective items were introduced by the phrase “Due to the current situation…”, which was referred to the Corona pandemic. Agreement or disagreement was scored on a 5-point scale (0—does not apply at all; 1—does not truly apply; 2—neither yes nor no; 3—applies quite a bit; 4—applies very much). The internal consistency of the 12-item version is good (Cronbach’s alpha = 0.82). The scores were referred to a 100% level (transformed scale score). Scores > 60% indicate higher agreement (positive attitude/behavior), scores between 40 and 60 indifference, and scores < 40 disagreement (negative attitude/behavior).

#### Well-being

To assess participants’ well-being, we used the WHO-Five Well-being Index (WHO-5). This short-scale avoids symptom-related or negative phrasings and measures well-being instead of the absence of distress (Bech et al. 2013). Representative items are “I have felt cheerful and in good spirits” or “My daily life has been filled with things that interest me”. Respondents assess how often they had the respective feelings within the last two weeks, ranging from at no time (0) to all of the times (5). Here we report both the sum scores ranging from 0 to 25 (scores < 13 would indicate depressive states) and also the scores referred to a 100% level.

#### Perception of burden and corona pandemic outcomes

Perceived daily life affections due to disease-related symptoms, feelings to be restricted in daily life by the pandemic, and feelings of being under pressure (i.e., stress and fear) due to the pandemic were measured with three numeric analogue scales (NAS), ranging from 0 (not at all) to 100 (very strong) as described (Büssing et al. 2020a). These three scores can be summarized as a “stressor” score with adequate internal reliability in this sample (Cronbach’s alpha = 0.78).

Several tumor patients reported that they were “Irritated or unsettled by different statements about the danger and the course of the corona infection in the public media” and that they are “Worrying to be infected with COVID-19 virus and to have complicated course of disease”. Both statements were addressed with two single items as described (Büntzel et al. 2020a; Büssing et al. 2020a). Agreement to these statements was scored from not at all, a little, somewhat and very much.

#### Meaning in life (MLQ)

Whether persons are in search for meaning in life or already have found it, was measured with the 10-item Meaning in Life Questionnaire (MLQ) (Steger et al. 2006). The 5-item Search subscale uses items such as “I am looking for something that makes my life feel meaningful” and “I am always looking to find my life’s purpose”, and the 5-item Presence subscale items such as “My life has a clear sense of purpose” and “I have discovered a satisfying life purpose.” Internal consistency of both subscales is good to very good (Cronbach’s alpha between 0.81 and 0.92). Items are scored form 1 (absolutely untrue) to 7 (absolutely true). The higher the MLQ subscale scores, the higher the perceived meaning in life is.

#### Indicators of spirituality

The SpREUK questionnaire was developed to investigate whether or not patients with chronic diseases living in secular societies rely on spirituality as a resource to cope (Büssing et al. 2005; Büssing 2010). The 15-item instrument differentiates 3 factors: (1) the *Search* factor deals with patients’ intention to find or have access to a spiritual/religious resource to cope with illness, and having an interest in spiritual/religious issues; (2) the *Trust* factor is a measure of intrinsic religiosity dealing with patients’ conviction to be connected with a higher source which carries through, and to be sheltered and guided by this source—whatever may happen; (3) the *Reflection* factor deals with cognitive reappraisal and subsequent attempts to change (i.e., reflect on what is essential in life; hint to change life; chance for development; illness has meaning, etc.). Some phrasings were moderately adjusted in the sense that the phrasing “my illness” (has made me to …) was replaced by “the current situation” (has made me to…). The scales’ internal consistency ranges from Cronbach’s alpha = 0.86 to 0.91. The items were scored on a 5-point scale from disagreement to agreement (0—does not apply at all; 1—does not truly apply; 2—neither yes nor no; 3—applies quite a bit; 4—applies very much). The scores were referred to a 100% level (transformed scale score). Scores > 60% indicate higher agreement (positive attitude), scores between 40 and 60 indifference, and scores < 40 disagreement (negative attitude).

We added a further item (A37) with the same scoring which asks whether faith is a “strong hold in difficult times”. This item was used as a differentiating variable in terms of a religious attitude. Frequency of meditation and prayer as actional aspects of spirituality were addressed with two items and scored on a 4-grade scale (never, at least once per month, at least once per week, at least once per day) as described (Büssing et al. 2020a).

The SpREUK includes two specific items which ask whether persons regard themselves as a spiritual and/or religious person (without defining what these terms may mean) (Büssing et al. 2005; Büssing 2010). Scores > 2 indicate agreement and scores < 3 indifference or disagreement. Subsequently one can categorize person who regard themselves as religious and spiritual (R + S +), religious but not spiritual (R + S−), not religious but spiritual (R−S +) and neither religious nor spiritual (R−S−).

#### Awe and gratitude (GrAw-7)

To address whether persons are able to perceive feelings of wondering awe in specific stations (mainly in the nature) with subsequent feelings of gratitude as a perceptive aspect of spirituality, we used the 7-item Awe/Gratitude scale (GrAw-7) (Büssing et al. 2018). This scale has good psychometric properties (Cronbach’s alpha = 0.82) and uses items such as “I stop and then think of so many things for which I'm really grateful”, “I stop and am captivated by the beauty of nature”, “I pause and stay spellbound at the moment” and “In certain places, I become very quiet and devout”. All items were scored on a 4-point scale (0—never; 1—seldom; 2—often; 3—regularly) and referred to a 100-point scale.

### Statistical analyses

Descriptive statistics, analyses of variance (ANOVA), generalized linear modeling and comparison of variables from both independent samples using the Whitney–Mann-*U* test were computed with SPSS 23.0 and R 4.0.2. Given the exploratory character of this study, significance level was set at *p* < 0.01.

## Results

### Description of the sample

The surveys were started by 330 persons in sample 1 (from wave 1) and 245 in sample 2 (from wave 2). Among them, 38 were not proceeding the survey in sample 1 (11.5%) and 24 in sample 2 (9.8%). Their basic data did not differ with respect to gender and age, while more proceeders were living with a partner (74% vs. 57%, *p* < 0.0001), were a-religious (39% vs. 18%, *p* = 0.001) and had a primary tumor (66% vs 46%, *p* = 0.010). All further analyses were performed with the sample of proceeding participants (Table 1).

**Table 1:** Description of the samples (*N* = 575)

	Participants first wave	Participants second wave	*p*-values (Pearson’s Chi^2^) (Mann–Whitney-*U* test)
Starter of the survey (*n*)	330	245	
Proceeding participants (%)	292 (88.5)	221 (90.2)	n.s
*Proceeding participants only*			
Mean age (years)	66.7 ± 10.8	66.0 ± 10.2	n.s
Gender (%)			n.s
Women	28.1	22.6	
Men	71.9	77.4	
Partner status (%)			n.s
With partner	19.5	25.8	
Without partner	80.5	74.2	
Tumor status (%)			
Primary tumor	67.1	65.2	n.s
Relapse	20.9	16.3	n.s
Metastases	22.6	12.7	0.004
Tumor stage III–IV (%)	42.8	22.2	< 0.0001
Treatment intention (%)			
Curative	28.1	21.3	n.s
Palliative	14.4	7.7	0.019
Not actively at the moment	10.3	14.5	n.s
Already effectively treated	47.3	47.5	n.s
COVID-19 testing (%)			
Negatively tested	5.8	31.7	< 0.0001
Not yet tested	92.1	62.0	< 0.0001
Irritated by statements about danger and course of the infection in public media (%)			n.s
Not at all	10.3	15.8	
A little	29.5	26.0	
Somewhat	34.8	37.7	
Very much	25.3	20.5	
Worrying to be infected and to have complicated course of disease (%)			n.s
Not at all	12.6	15.6	
A little	30.8	29.8	
Somewhat	32.2	33.9	
Very much	24.5	20.6	
SpR self categorization (%)			0.010
R + S +	16.0	6.6	
R + S−	17.2	15.2	
R−S +	6.3	5.6	
R−S−	60.5	72.7	
Faith as a strong hold (%)			n.s
No	51.4	60.7	
Indifferent	16.1	16.4	
Yes	32.5	22.9	
Reaction due to the pandemic			
Lost my faith (%)	6.2	6.2	n.s
More interest in SpR (%)	13.7	9.9	n.s

In both samples, we had a dominance of male persons which is due to the tumor specialization of recruiting institutions. Patients with prostate cancer (37%), larynx tumors (17%) and nasal/paranasal tumors (12%) were predominating; further tumors were breast cancer (9%), rectal cancer (3%) and various other (22%). Patients’ gender, mean age and partner status did not significantly differ between both recruiting waves. However, in sample 2, the proportion of persons with higher tumor stages and metastases was significantly lower (10% lower, *p* = 0.004). All further sociodemographic and disease-related details can be found in Table 1.

### Stressors and wellbeing

The proportion of persons who stated that they are “Irritated by statements about danger and course of the infection in public media” and who are “Worrying to be infected and to have complicated course of disease” was not significantly different between both samples (Table 1).

With respect to the stressors, the proportion of persons who stated daily life affections due to their tumor symptoms or who felt under pressure due to the pandemic was similar, while the proportion of tumor patients who stated to feel restricted in their daily life concerns due to the pandemic was significantly lower in the second wave sample, but only weakly (Cohen’s *d* = 0.24) (Table 2). Patients’ wellbeing and meaning in life scores were similar (Table 2). The proportion of persons with wellbeing scores < 13 (indicating depressive states) was decreasing from 35 to 31%, while the proportion of persons with high wellbeing (WHO-5 scores > 19) was similar (Table 1).

**Table 2:** Differences of attitudes, perceptions and behaviors between tumor patients from the first and the second wave

	Participants first wave	Participants second wave	*p*-values (Mann–Whitney-*U* test)	Effect sizes (Cohen’s *d*)
Stressors (NAS 1–3)	38.7 ± 22.5	35.5 ± 22.7	n.s	
Daily life affection due to symptoms (NAS1)	39.8 ± 26.4	39.2 ± 27.7	n.s	
Restricted in daily life by the pandemic (NAS2)	45.1 ± 26.4	36.7 ± 26.1	< 0.0001	0.24
Under pressure due to the pandemic (NAS3)	32.1 ± 28.5	30.8 ± 26.5	n.s	
Wellbeing (WHO-5)				
100% score	58.6 ± 24.0	59.4 ± 24.3	n.s	
Sum score	14.7 ± 6.0	14.8 ± 6.1	n.s	
Sum score < 13 (%)	35.3	30.5	n.s	
Sum scores 13–18 (%)	31.8	36.7		
Sum score > 19 (%)	32.9	32.9		
Awe/gratitude (GrAw-7)	57.2 ± 20.0	52.7 ± 21.3	0.039	0.22
Spirituality and coping (SpREUK-15)				
Search	25.6 ± 25.9	20.3 ± 25.4	0.008	0.21
Trust	38.9 ± 30.5	29.2 ± 30.3	< 0.0001	0.32
Reflection	45.6 ± 25.0	41.7 ± 25.4	n.s	0.15
Spiritual practices				
Meditation [0–3]	0.6 ± 1.0	0.4 ± 0.9	0.023	0.21
Praying [0–3]	0.9 ± 1.2	0.6 ± 1.1	0.006	0.26
Meaning in life (MLQ)				
Search	16.2 ± 7.7	16.7 ± 7.4	n.s	
Presence	26.7 ± 6.3	26.0 ± 6.4	n.s	
Perceived changes related to the pandemic (PCQ)				
Perception of nature and silence	58.9 ± 22.9	55.4 ± 24.1	0.060	0.15
More walks in nature (item t4)	2.50 ± 1.14	2.52 ± 1.22	n.s	
Worrying reflections and loneliness	50.1 ± 23.8	44.4 ± 24.8	0.008	0.24
Interest in spirituality	26.6 ± 29.6	22.0 ± 28.1	0.072	0.16
Intense relationships	59.0 ± 23.1	56.0 ± 27.4	n.s	

### Perceived changes due to the pandemic

Perceived changes in perceptions, attitudes and behaviors due to the pandemic scored significantly lower in wave 2 with respect to *Worrying reflections and loneliness* (Cohen’s *d* = 0.24) (which is rather moderately expressed), in trend for *Interest in spirituality* (Cohen’s *d* = 0.16) (which is still scored rather low) and *Perception of nature and silence* (Cohen’s *d* = 0.15) (which scores somewhat higher in general), but not significantly different for changes in terms of more *Intense relationships* (which scored rather high) (Table 2). All observed changes are small only.

### Indicators of spirituality

Although the proportion of persons who state that they have faith as a stronghold in difficult times was lower in the second wave, this difference is not significant (Table 1). However, the spiritual-religious self-categorization showed a small significant difference which is due to a higher proportion of R−S− persons (12% more) and a lower proportion of R + S + persons (~ 10% less). Nevertheless, the proportion of tumor patients who stated that they have lost their faith because of the pandemic was identically low (Table 1).

With respect to further indicators of spirituality, Awe/Gratitude (Cohen’s *d* = 0.22), religious Trust (Cohen’s *d* = 0.32), and the frequency of praying (Cohen’s *d *= 0.21) and meditation (Cohen’s *d* = 0.26) were significantly lower in wave 2’s patients, while Search for a spiritual source of help and Reflection of own disease and life concerns were lower too (Cohen’s *d* = 0.15), but not significantly (Table 2). However, the differences are small only.

Because the proportion of R−S− persons was larger in the second wave sample, we performed post hoc analyzes with SpR + persons only (*n* = 155) and found that the differences between both samples with respect to religious Trust became non-significant (66.0 ± 22.2 vs 65.6 ± 21.7, n.s.), while Awe/Gratitude (66.7 ± 19.5 vs 60.3 ± 18.4, *p* = 0.028; Cohen’s *d* = 0.33) and also *Perception of nature and silence* remained significantly different (61.6 ± 22.9 vs 52.3 ± 24.1, *p* = 0.015; Cohen’s *d* = 0.44). Further, the difference in the perception to be restricted in daily life by the pandemic (NAS2, 46.4 ± 28.4 vs 40.2 ± 27.4; n.s.) and *Worrying reflections and loneliness* became non-significant (52.1 ± 22.9 vs 45.9 ± 23.3; n.s.), too.

### Spirituality as a resource to cope

Because the aforementioned findings would indicate that some tumor patients may rely on their spirituality/faith as a resource to cope, we used “faith as a strong hold” as a differentiating variable in both samples. As shown in Table 3, persons who have faith as a strong hold scored stronger on indicators of spirituality (Awe/Gratitude, Search, Reflection, *Interest in Spirituality*), but also on perceived changes due to the pandemic, such as *Perception of nature and silence* and *Worrying reflections and loneliness*. Interestingly, those who do not have this resource, perceived restrictions in daily life due to the pandemic slightly lower, too. However, their wellbeing and *Relationships* did not significantly differ.

**Table 3:** Stressors, resources and perceived changes differentiated for “Faith as a strong hold” in both samples (wave 1 and wave 2)

Differentiating variable: faith as a strong hold in difficult times	Stressors (NAS1-3)	Daily life affection due to symptoms (NAS1)	Restricted in daily life by the pandemic (NAS2)	Under pressure due to the pandemic (NAS3)	Wellbeing (WHO-5 sum)	Wellbeing (WHO-5 100)	Meaning in life: presence	Meaning in life: search	Awe/gratitude	Search	Trust	Reflection	Perception of nature and silence	Worrying reflections and loneliness	Interest in spirituality	Intense relationships
No (*n* = 253)																
Mean	35.0	37.9	38.2	29.5	14.8	59.1	26.0	16.1	48.9	9.4	14.0	33.9	53.1	42.9	10.6	55.8
SD	21.7	27.0	25.4	26.5	6.0	24.1	6.6	7.6	18.8	15.7	17.0	23.5	23.7	24.8	19.3	26.3
Indifferent (*n* = 74)																
Mean	42.3	43.8	47.5	36.0	13.9	55.5	26.1	16.6	56.6	32.8	43.8	49.8	60.3	52.2	29.5	62.3
SD	23.0	26.1	28.3	29.9	6.1	24.4	5.5	7.2	21.4	23.0	16.8	19.6	21.2	23.0	28.7	21.3
Yes (*n* = 129)																
Mean	38.9	40.2	44.3	33.3	15.4	61.6	27.8	17.7	67.5	45.2	69.5	60.5	62.1	51.3	47.5	58.2
SD	24.7	28.0	27.6	29.2	5.6	22.5	5.5	7.7	17.8	26.1	22.3	21.6	23.2	23.0	29.0	24.2
All persons (*n* = 454)																
Mean	37.3	39.5	41.4	31.6	14.8	59.2	26.5	16.6	55.4	23.2	34.4	43.9	56.8	46.8	24.0	57.5
SD	22.9	27.1	26.7	27.9	5.9	23.8	6.2	7.6	20.6	25.9	30.6	25.3	23.5	24.4	28.9	25.0
*F*-value	3.3	1.4	4.4	1.8	1.5	1.5	3.7	1.8	**40.7**	**141.1**	**387.7**	**63.1**	**7.5**	**7.6**	**102.4**	2.0
*p*-value	0.038	n.s	0.013	n.s	n.s	n.s	0.027	n.s	** < 0.0001**	** < 0.0001**	** < 0.0001**	** < 0.0001**	**0.001**	**0.001**	** < 0.0001**	n.s

*p* values < 0.01 are highlighted in bold

### Multivariate generalized regression models

As there were some differences with respect to attitudes, perceptions, and behaviors between persons of samples 1 and 2, multiple regression analyses were performed to clarify influencing (independent) variables, particularly the effect of the respective time period (“wave”). We focused on the (dependent) variables *Perception of nature and silence* and *Worrying reflections and loneliness*, Awe/Gratitude and religious Trust. For that purpose, generalized linear models for analysis of variance to investigate the multivariate relationships between constructs were used. This model allows the use of both categorical and numerical independent variables to explain the variance inherent in the model observed for the dependent variable. Results are displayed in Table 4. Variable selection was performed through stepwise selection based on Akaike Information Criteria (AIC).

**Table 4:** Results of the generalized regression models for analysis of variance

Models	DF	*F*-value	*p*-value
*Dependent variable: perception of nature and silence (PCQ-12)*			
Independent variables			
Stressors	1	5.34	0.02
Awe/gratitude	1	73.99	< 0.001
Reflection	1	17.47	< 0.001
Non-religious	1	4.12	0.04
Worrying reflections and loneliness	1	60.68	< 0.001
Interest in spirituality	1	7.83	0.005
Intense relationships	1	45.75	< 0.001
*Dependent variable: model for worrying reflections and loneliness (PCQ-12)*			
Independent variables			
Wave	1	12.89	< 0.001
Stressors	1	200.67	< 0.001
Meaning in life search	1	105.26	< 0.001
Search	1	7.55	0.006
Reflection	1	13.53	< 0.001
Perception of nature and silence	1	51.06	< 0.001
Interest in spirituality	1	32.33	< 0.001
Depressive state	1	9.86	< 0.001
*Dependent variable: model for awe/gratitude (GrAw-7)*			
Independent variables			
Wave	1	9.24	0.002
Faith as hold	2	36.01	< 0.001
Reflection	1	54.9	< 0.001
Trust	1	10.3	0.001
Perception of nature and silence	1	26.5	< 0.001
Wellbeing (WHO5-100)	1	12.3	< 0.001
*Dependent variable: model for religious trust (SpREUK-15)*			
Independent variables			
Well-being (WHO5-100)	1	11.05	< 0.001
Awe/gratitude	1	382.02	< 0.001
Faith as hold	2	457.01	< 0.001
Search	1	216.34	< 0.001
Reflection	1	11.6	< 0.001
Non-religious	1	39.52	< 0.001

The final model for *Worrying reflections and loneliness* was significantly defined by the time of recruitment (wave), by Stressors, Search, Reflection, Meaning in Life: Search, *Perception of Nature and Silence*, *Interest in Spirituality* and depressive symptoms (WHO-5 score < 13); despite Search having lower scores in wave 2, Meaning in Life: Search has increased; scores for Reflection and *Interest in Spirituality* decreased both.

Changes in patients’ *Perception of nature and silence* were significantly influenced by Awe/Gratitude, Reflection, *Worrying reflections and loneliness, Interest in Spirituality* and *Intense relationships,* and by being non-religious (R−S−) and by perceived stressors. Here, the strongest effect was due to Awe/Gratitude, while the time of recruitment had no significant influence.

The model for Awe/Gratitude shows a significant relationship with time of recruitment (wave). The second wave displays lower values for faith as a strong hold, lower scores for Reflection, religious Trust and *Perception of Nature and Silence,* but higher values for well-being. For religious Trust, which was lower in wave 2’s patients was not influenced by the time of recruitment (wave), but by lower Awe/Gratitude, lower faith as a strong hold, Search and Reflection.

## Discussion

Post-COVID-19 stress disorders were themed as an emerging consequence of the actual pandemic. To avoid psychiatric disorders in the future, the baseline mechanism of patients’ fears and coping strategies have to be better understood (Tucker and Czapla 2021). During the months of March and April 2020, Germany was in a lockdown and most avoided social contacts and kept their distance from others to avoid infection with the COVID-19 virus. Then during the summer months, the number of infected persons and death rates declined again and most were delighted that the situation seemed to improve. At that time there were repeated demonstrations by ‘Corona Deniers’ and persons who refused to wear a face-mask (Sweet 2020; Reuters 2020) arguing that all protective measures of the government would have been exaggerated and that the situation was not that bad at all. There was a clear societal divide, those who denied the COVID-19 risk and refused protection measures and those who pressed for more security and protection, particularly for groups at risk. During October the numbers of infected persons were obviously increasing again, with more than 10,000 infected persons per day during November, and in January 2021 more than 20,000 new infected persons per day. During the second wave, for tumor patients one may assume two different scenarios: (1) they might be more stable because they have learned to cope with the pandemic; (2) they are still insecure and in fear of the outcomes and course of the pandemic.

Comparing both samples from wave 1 and wave 2 of the pandemic revealed that a large proportion of tumor patients is still “irritated by statements about danger and course of the infection in public media” (60% vs 58%) and are “worrying to be infected and to have a complicated course of disease” (58% vs 55%). Their fears and worries were not significantly different, with the exception of a lower perception of being restricted in daily life by the pandemic at the start of wave 2 patients. Their stressors seem to be similar, but it might be that the ‘summer lightness’ (with a decrease of pandemic restrictions, decrease of number of infected and dying persons) decreased their perception of being restricted, too. In fact, the second lockdown followed in December 2020. However, also (non-tumor) persons recruited in June 2020 were “irritated by statements about danger and course of the infection in public media”, but to a lower extend (44%) (Büssing et al. 2020b). In contrast, Musche et al (2020) reported that tumor patients had no higher distress scores or anxiety compared to healthy persons, while tumor patients had more adherence safety behavior (indicating that they are more aware of their risks) and more dysfunctional safety behaviors (indicating that they nevertheless are afraid of the unpredictable pandemic outcomes). Musche et al. (2020) concluded that “specific interventions are needed to prevent anxiety and improve mental health during the COVID-19 pandemic”.

With respect to perceived changes due to the pandemic which can be interpreted in part as a reappraisal coping strategy or in terms of posttraumatic growth (Büssing et al. 2020a), one may find a similar direction in terms of lower *Worrying reflections and loneliness,* while there were lower scores for the relatively high *Perception of nature and silence* in trend only, indicating a return to ‘normality’—although their walks in nature (item t4) were similar and not decreasing. More *Intense relationships* were perceived similarly high in both samples. Also tumor patients’ wellbeing was similar, although the fraction of persons with low wellbeing scores were slightly improving. It seems that patients have noticed that the second wave of the pandemic has started and that the risks are the same, and thus the stressors are similar and perceived changes due to the pandemic, too.

It was an interesting finding that some inter-group differences seemed to be influenced by the proportion of R−S− persons in the samples, particularly perception to be restricted in daily life by the pandemic, *Worrying reflections and loneliness*, while Awe/Gratitude and also *Perception of nature and silence* remained significantly different. Further analyses revealed that those who can rely on their faith as a strong hold, have—apart from higher spirituality scores—stronger *Perception of nature and silence* and are more aware of specific situations in terms of wondering awe and subsequent feelings of gratitude. However, they are also more concerned with worrying reflections. Having such a resource does not prevent such worries. Multiple regression models revealed that the lower scores of *Worrying reflections and loneliness* in sample 2 can be assigned to time (beginning wave 2), but also to a mix of stressors and available resources (e.g. Search, Reflection, Meaning in Life, *Perception of Nature and Silence*, *Interest in Spirituality*). The lower scores for *Worrying Reflections and Loneliness* can mostly be explained by a decrease of perceived stressors and increase of patients’ search for meaning in life. In contrast, the lower scores of *Perception of nature and silence* during wave 2 can be ascribed to both, a higher proportion of R−S− patients in sample 2 and to related differences in perceptions and behaviors. These perceived changes were significantly influenced by indicators of spirituality (e.g. Awe/Gratitude, Reflection, *Interest in Spirituality*)*,* perceived stressors and *Worrying reflections and loneliness*, while the time of recruitment had no significant influence in the respective regression model. In contrast, the decrease of perceptions of wondering awe and gratitude were significantly related to the time of recruitment (wave). In fact, patients in the starting second wave had less faith as a strong hold, lower religious Trust and lower *Perception of Nature and Silence,* and this may have contributed to a lower openness (or even ability) to perceive the ‘wonder of the moment’ despite of the pandemic. Tumor patients lower religious Trust in sample 2 was not related to time in general, but due to lower perception the sacred in patients’ life concern (Awe/Gratitude), lower Search for spiritual resources to cope, and lower Reflection of life concerns—all indicators of spirituality. Here, wellbeing might be a booster and could positively improve religious Trust, which is negatively related to being R−S−.

## Limitations

This study has a cross-sectional design which does not allow causal conclusions. Patients were enrolled in the same recruiting centers, but sample 2 patients are not necessarily the same as in sample 1. Thus, both samples were regarded as independent samples. All changes are thus either due to differences in the sample proportion or due to changes in attitudes and behaviors within time. To account for this, we performed multiple regression analysis with significantly different variables as dependent variables and recruitment time (wave) as an independent variable. Further, due to the treatment specialties of the recruiting institutions we have a dominance of male patients. However, perceived changes (PCQ) were not different between women and men (Büssing et al. 2020a).

## Outlook

For all of us it is necessary to learn from the experiences of the first wave of the COVID-19 pandemic and the months after, because personal and societal resources are limited. Health professionals need strategies to use all possible resources and interventions to compensate for described deficits in the oncological service system during the pandemic. Self-help, and self-coping are important factors for optimized care.

During the first wave of the COVID-19 pandemic, we noticed that patients perceived positive and negative changes of their attitudes and behaviors. Their fears and worries also resulted in higher sensitivity and emotional stress of the patients, too. Emotional stress is usually limited by time and stimulus intensity. However, during the actual second wave a limitation of stress stimuli was obviously not developed because of on-going repeated appeals to avoid social contacts on the one hand, and more strongly increasing numbers of infected persons and death rates on the other hand. Described coping strategies have to be adapted prospectively and should be used deliberately.

For oncologists and psychologists, it is important to know that their patients’ situation has not changed within the time of the pandemic and that they still require information, close support, and encouragement to rely on their resources to cope. Even when they may not share patients’ religious believes, these may be a resource that influences how they perceive their current situation in times of the pandemic, and which resource they may utilize. Particularly mindful awareness of nature, times of silence and reflections are important to avoid that their thoughts are trapped in negative loops and thus have an alternative approach to focus on the still positive aspects in their life. For that purpose, training to mindful awareness might be helpful to facilitate wondering awe and subsequent feelings of gratefulness—despite the restrictions. As discussed, awe/gratitude are among the best predictors of perceived changes due to the pandemic and contribute to a person’s wellbeing (Büssing et al. 2020b). Others reported that such feelings of awe are related to a sense of connection and belonging (Prade and Saroglou 2016), which can be seen as a stabilizing resource, too. This could be encouraged in times of social distance.

At the end, we come back to the data of German Cancer Comprehensive Centers in 2020. Non-medical care was restricted at first. We encourage all colleagues to correct this point. During the pandemic, ‘mental care’ will help to overcome the fears and to improve wellbeing of patients as well health care professionals. Whether such support can also prevent some patients’ intention to avoid conventional treatments and to adhere to alternative treatments, which often are more attentive and conversational, is unclear. Disappointment about a lack of oncologists’ explanations and communication may raise patients’ interest in alternative therapeutic approaches even in non-Corona times (Schallock et al. 2019). At least one has to state that several patients have considered stopping their conventional treatment during the first wave of the pandemic (Büntzel et al. 2021).
